# Ribosome Profiling Reveals HSP90 Inhibitor Effects on Stage-Specific Protein Synthesis in *Leishmania donovani*

**DOI:** 10.1128/mSystems.00214-18

**Published:** 2018-11-20

**Authors:** Eugenia Bifeld, Stephan Lorenzen, Katharina Bartsch, Juan-José Vasquez, T. Nicolai Siegel, Joachim Clos

**Affiliations:** a Leishmaniasis Group, Bernhard Nocht Institute for Tropical Medicine, Hamburg, Germany; b Research Center for Infectious Diseases, University of Würzburg, Würzburg, Germany; c Bioinformatics Unit, Bernhard Nocht Institute for Tropical Medicine, Hamburg, Germany; d Department of Veterinary Sciences, Experimental Parasitology, Ludwig Maximilians University, Munich, Germany; e Biomedical Center Munich, Department of Physiological Chemistry, Ludwig-Maximilians-Universität, Planegg-Martinsried, Germany; University of California, Berkeley

**Keywords:** HSP90, *Leishmania donovani*, radicicol, ribosome profiling

## Abstract

The 90-kDa heat shock protein (HSP90) of eukaryotes is a highly abundant and essential chaperone required for the maturation of regulatory and signal proteins. In the protozoan parasite Leishmania donovani, causative agent of the fatal visceral leishmaniasis, HSP90 activity is essential for cell proliferation and survival. Even more importantly, its inhibition causes life cycle progression from the insect stage to the pathogenic, mammalian stage. To unravel the molecular impact of HSP90 activity on the parasites’ gene expression, we performed a ribosome profiling analysis of L. donovani, comparing genome-wide protein synthesis patterns in the presence and absence of the HSP90-specific inhibitor radicicol and an ectopically expressed radicicol-resistant HSP90 variant. We find that ribosome-protected RNA faithfully maps open reading frames and represents 97% of the annotated protein-coding genes of L. donovani. Protein synthesis was found to correlate poorly with RNA steady-state levels, indicating a regulated translation as primary mechanism for HSP90-dependent gene expression. The results confirm inhibitory effects of HSP90 on the synthesis of *Leishmania* proteins that are associated with the pathogenic, intracellular stage of the parasite. Those include heat shock proteins, redox enzymes, virulence-enhancing surface proteins, proteolytic pathways, and a complete set of histones. Conversely, HSP90 promotes fatty acid synthesis enzymes. Complementing radicicol treatment with the radicicol-resistant HSP90rr variant revealed important off-target radicicol effects that control a large number of the above-listed proteins. *Leishmania* lacks gene-specific transcription regulation and relies on regulated translation instead. Our ribosome footprinting analysis demonstrates a controlling function of HSP90 in stage-specific protein synthesis but also significant, HSP90-independent effects of the inhibitor radicicol.

**IMPORTANCE** *Leishmania* parasites cause severe illness in humans and animals. They exist in two developmental stages, insect form and mammalian form, which differ in shape and gene expression. By mapping and quantifying RNA fragments protected by protein synthesis complexes, we determined the rates of protein synthesis for >90% of all *Leishmania* proteins in response to the inhibition of a key regulatory protein, the 90-kDa heat shock protein. We find that *Leishmania* depends on a regulation of protein synthesis for controlling its gene expression and that heat shock protein 90 inhibition can trigger the developmental program from insect form to mammalian form of the pathogen.

## Abstract (precis)

*Leishmania* parasites cause severe illness in humans and animals. They exist in two developmental stages, insect form and mammalian form, which differ in shape and gene expression. By mapping and quantifying RNA fragments protected by protein synthesis complexes, we determined the rates of protein synthesis for >90% of all *Leishmania* proteins in response to the inhibition of a key regulatory protein, the 90-kDa heat shock protein. We find that *Leishmania* depends on a regulation of protein synthesis for controlling its gene expression and that heat shock protein 90 inhibition can trigger the developmental program from insect form to mammalian form of the pathogen.

## INTRODUCTION

All living organisms express a group of proteins known as heat shock proteins (HSPs) that function as molecular chaperones, assisting newly synthesized, translocated, or stress-damaged proteins in attaining their native and functional state and preventing the harmful intracellular aggregation of proteins. Apart from this well-established role, heat shock proteins are increasingly identified as part of regulatory pathways. In eukaryotes, the 90-kDa class of HSPs (HSP90), assisted by a wide array of chaperones and cochaperones, is well known to affect cellular differentiation, gene expression control, and signal transduction pathways. The over 200 client proteins identified to date include signal transduction protein kinases, nuclear receptors, promoter- and enhancer-binding transcription factors, and cytoskeletal proteins (1–5). HSP90 is also involved in the regulation of its own synthesis by its interaction with heat shock transcription factor (6, 7).

Nonpathogenic and pathogenic microorganisms also depend on HSP90 for differentiation and cell cycle control. In Saccharomyces cerevisiae, 10 to 20% of the proteome is under HSP90-dependent control (5, 8). In the malaria-causing parasite Plasmodium falciparum, HSP90 is essential for growth and development in human blood cells (9–11). Similar findings were reported for the related apicomplexan parasite Toxoplasma gondii (12) and for the gut parasite Entamoeba histolytica (13). HSP90 is also essential in Leishmania donovani, the causative agent of the lethal kala-azar fever, a.k.a. visceral leishmaniasis, where it was shown to be essential for proliferation and intracellular, parasitic persistence (14, 15).

The genus *Leishmania* is transmitted by phlebotomine sandflies to a broad range of vertebrates, including canines, rodents, and humans. The parasites reside in macrophages and other cells of the reticuloendothelial system, where they multiply within parasitophorous vacuoles that are derived from phagosomes (16–18). This mammalian parasite stage, the amastigote, differs from the insect stage, the promastigote, not only morphologically but also metabolically (19–21), since the transmission from a poikilothermic arthropod host to a homeothermic mammalian host requires the parasites to adapt to elevated temperatures, an acidic milieu, and alternative carbon sources.

Classic *cis*-regulatory gene promoter and enhancer elements are absent from the *Leishmania* genome. In addition, *Leishmania* genes are organized in multigene, unidirectional transcription units (22), giving rise to multicistronic mRNA precursors which are processed into monocistronic mRNAs by coupled transsplicing and polyadenylation (23). Combined, these findings rule out a control of individual genes at the level of transcription (24–26), pointing at RNA stability (27–29) and translation control (30, 31) as regulated steps of gene expression. Yet, correlation between the steady-state levels of mRNAs and their corresponding proteins is only between 20 and 40% (32), arguing against RNA processing and/or stability as the dominating control mechanisms. Although lacking inducible transcription, the leishmaniae respond to elevated temperature and other stresses with the increased synthesis of HSPs (30, 31).

The *Leishmania* HSP families comprise members that are expressed constitutively during both life cycle stages, e.g., HSP70 and HSP90 (30), and others whose expression increases during the conversion into the amastigote stage (21, 33, 34). The *in vitro* conversion from elongated, flagellated L. donovani promastigotes to ovoid, aflagellated, so-called axenic amastigotes can be achieved by the elevation of the culture temperature to 37°C and the acidification of the growth medium (35). The same morphological differentiation can be observed when L. donovani is treated with the HSP90-specific inhibitors geldanamycin (GA) or radicicol (RAD), which both target the special ATPase domain of HSP90 chaperones. These parasites also show an amastigote-like morphology and an increased expression of the amastigote-specific A2 protein family (14). This points to a central role for HSP90 in the parasite’s life cycle and stage conversion. In *Leishmania*, HSP90 (synonymously called HSP83) is encoded by multiple tandemly arranged gene copies (27, 36), and is a highly abundant, constitutively expressed protein in *Leishmania* spp. (30). It interacts with chaperones such as HSP70 and various cochaperones to form so-called foldosome complexes (37). Both GA and RAD bind HSP90 and inhibit its ATPase domain, thereby abrogating foldosome activity and causing cell growth arrest (38). A single amino acid exchange in the ATP-binding pocket of HSP90 can abrogate the RAD-mediated inhibition. This was first observed in the RAD-producing fungus Humicola fuscoatra (39) and correlated with an isoleucine residue in place of a highly conserved leucine. Exchange of the corresponding Leu33 against Ile in the L. donovani HSP90 and overexpression of this transgene prevent RAD-induced growth arrest and RAD-triggered amastigote differentiation (15), completely reversing the effects of HSP90 inhibition. However, a putative endoplasmic reticulum-specific HSP90 family member, GRP94 (40), and the supposed mitochondrial Trap1/HSP75 chaperone (41, 42) both contain ATPase domains that are homologous to that of HSP90 and may also be targeted by ATP competitors.

To analyze stage-specific gene expression at the relevant level, i.e., protein synthesis and abundance, proteome comparisons have been used, producing insight into the changes incurred by the parasite during stage conversion. A detailed analysis of the proteomic changes in the course of axenic, temperature-induced promastigote-to-amastigote conversion (21) confirmed earlier assessments for single proteins but also showed a “retooling of metabolic pathways” occurring during the differentiation. L. donovani undergoes a shift from carbohydrate metabolism pathways in the insect host to using fatty acids and amino acids as carbon sources while residing intracellularly in the mammalian host, reflecting the changing availability of nutrients. In spite of their advantages, such as detection of posttranslational modifications, comparative proteomics have limitations. Mass spectrometric detection of peptides requires high-picomolar quantities and is biased toward ionizable peptides, reducing the overall detection to ∼20% of the hypothetical proteome, even with advanced technologies (43).

Ribosome profiling is a new technique related to the DNase footprint assay (44) and able to fill the knowledge gap between RNA abundance and proteome analysis. It was developed by Ingolia et al. (45) and is based on the deep sequencing of ribosome-protected mRNA fragments. The comparison of the deep sequencing data from ribosome-protected mRNA fragments and the total mRNA from a cell allows distinguishing mRNA that is actively translated at a given time point in the cell, but also the translation efficiency, i.e., the number of ribosome-protected RNA fragments for each given mRNA. This facilitates quantification of nascent protein synthesis and identification of new and unusual open reading frames (ORFs) or short regulatory upstream ORFs (uORFs) in the untranslated regions (46, 47). This is of high importance for the study of organisms whose gene expression control relies on posttranscriptional events only, e.g., the Trypanosomatida (29). Ribosome profiling was used to unravel the stage conversion pathways in Trypanosoma cruzi (48), T. brucei (49, 50), and Toxoplasma gondii (51). In the second study, ribosome profiling also revealed new coding sequences that had escaped previous, algorithm-based detection and annotation. Since small proteins often escape detection by mass spectrometry due to not yielding enough peptides for identification, ribosome profiling can detect expression of short ORFs more sensitively.

In this paper, we applied ribosome profiling to monitor the changes in the protein synthesis induced by HSP90 inhibition of L. donovani parasites. HSP90 activity or the lack thereof affects the synthesis of several chaperone proteins, but also of histones, amastins, proteolytic proteins, and redox enzymes. We also find evidence that the HSP90 inhibitor radicicol has significant off-target effects, i.e., non-HSP90-specific effects, that modulate the synthesis of proteins. We find that the observed changes of protein synthesis do not correlate with changes in RNA abundance, confirming earlier findings obtained by RNA arrays and comparative proteomics (32).

## RESULTS

### Ribosome footprints faithfully map open reading frames of L. infantum.

**FIG 1:**
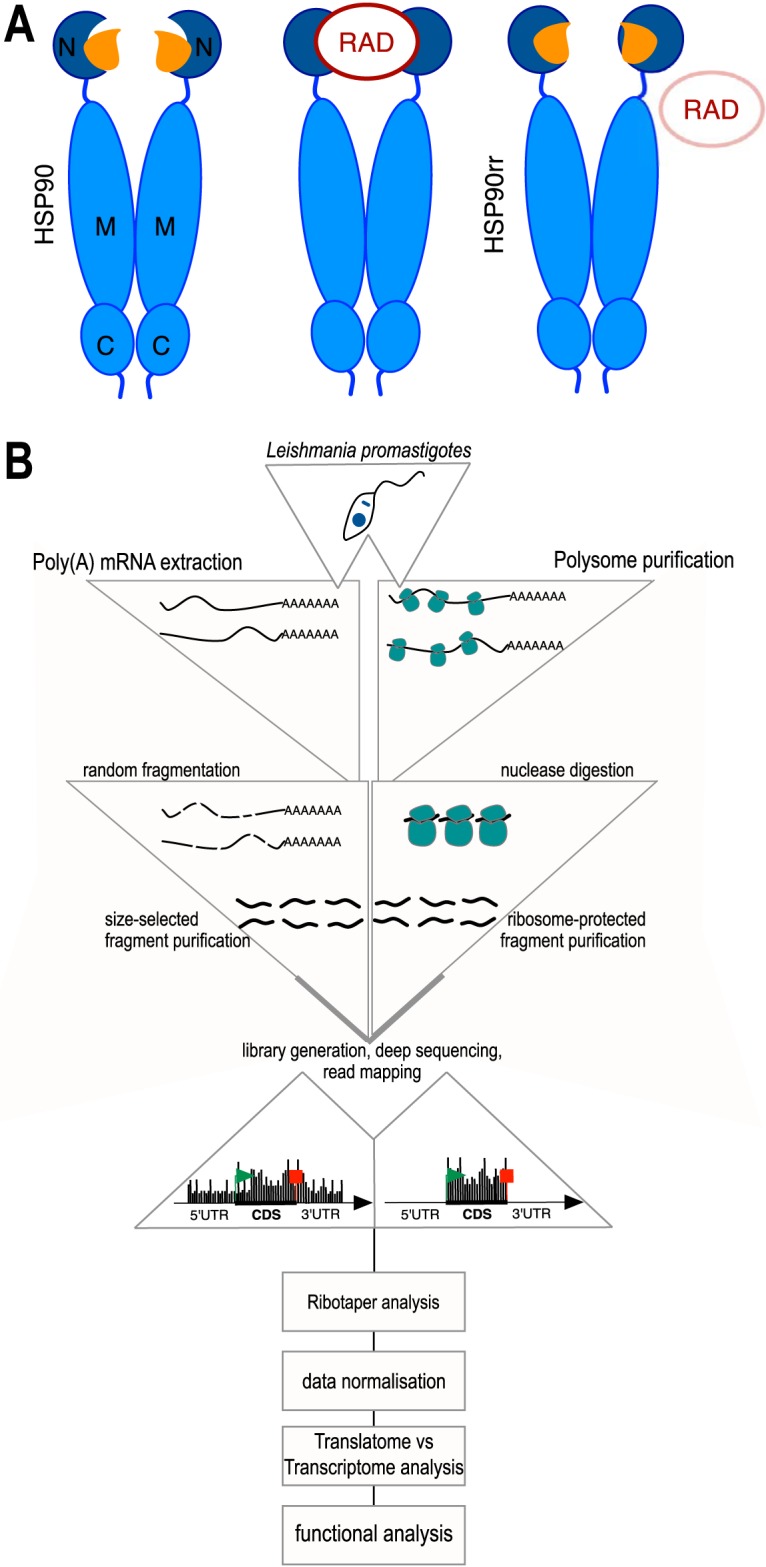
Experimental setup. (A) Schematic representation of biological samples used in the analysis showing (i) HSP90 able to bind ATP (orange), (ii) HSP90 bound by RAD, and (iii) HSP90rr able to bind ATP, no binding of RAD. N, N-terminal domain; M, middle domain; C, C-terminal domain. (B) Flow chart of ribosome profiling analysis.

The first aim of our study was to assess the accuracy of ribosome profiling for *Leishmania* promastigotes. For this we used three different promastigote populations: (i) wild-type L. donovani in the absence (WT-RAD) and (ii) presence (WT+RAD) of RAD at its IC_80_, and (iii) the RAD-resistant strain L. donovani [Hsp90rr] (15) in the presence of RAD (HSP90rr+RAD) (Fig. 1A). As expected, *in vitro* growth of WT+RAD is reduced by 75% compared with WT-RAD, whereas HSP90rr+RAD shows a 35% growth reduction, confirming that HSP90rr confers RAD resistance (see Fig. S1 in the supplemental material).

10.1128/mSystems.00214-18.1 FIG S1 Affirmation of IC_50_. Relative growth of WT-RAD cells (100%), WT+RAD (IC_80_), and HSP90rr+RAD (IC_80_) after 3 days. Download FIG S1, PDF file, 0.05 MB. Copyright © 2018 Bifeld et al. 2018 Bifeld et al. This content is distributed under the terms of the Creative Commons Attribution 4.0 International license.

Parasites from those populations were collected and treated with cycloheximide and heparin to arrest ribosomes *in situ*. Cells were then lysed, and the lysates were split equally for (i) isolation of total mRNA or (ii) RNase treatment followed by monoribosome purification using a sucrose density gradient centrifugation and a UV-light absorbance-based fractionation (Fig. S2). The protected RNA (ribosome footprints) was extracted from the monosomes while the total mRNA was chemically fragmented according to established protocols to yield ribosome footprint-comparable fragments (50, 52, 53). Nucleic acid fragments from both fractions were then processed for next-generation sequencing. Resulting sequence reads were then aligned to the L. infantum reference genome, which is >99% identical to the L. donovani genome, but offers better coverage and annotations. We used the RiboTaper analysis pipeline (54) to map the genome-wide distribution of protected sites (P sites), a.k.a. ribosome footprints (RFPs) (Fig. 1B) and to quantify P site alignments to coding sequences (CDSs). The latter were used as a measure of the translation rate of a CDS.

10.1128/mSystems.00214-18.2 FIG S2 Sample preparation. Optical density detection of the monosome fraction. The fraction was monitored using a Gradient Station (Science Services) at *A*_254_ (*y* axis) through the length (distance in mm) of the sample tube (*x* axis). The first peak between 0 and 20 mm represents the free RNA fraction. Fractions representing the monosome peak were collected at approximately a 50-mm distance from the top of the sucrose gradient. The three samples are shown in different colors as depicted. Download FIG S2, PDF file, 0.5 MB. Copyright © 2018 Bifeld et al. 2018 Bifeld et al. This content is distributed under the terms of the Creative Commons Attribution 4.0 International license.

Of the >98 million reads obtained from the three samples, 32.5 million reads aligned to the L. infantum protein coding sequences, with 59.7 million reads aligning to rRNA sequences. Of the 8,237 protein coding sequences identified in the L. infantum genome, >97% and ∼99% were represented by RFP reads and RNA reads, respectively (Table S1). The RNA-Seq and P site alignment per CDS as raw and normalized values, as well as the calculated translation efficiency (TE) values calculated, are shown in Table S2 (WT-RAD), Table S3 (WT+RAD), and Table S4 (HSP90rr+RAD).

10.1128/mSystems.00214-18.3 TABLE S1 Primary analysis of NGS sequence reads. Download Table S1, XLSX file, 0.01 MB. Copyright © 2018 Bifeld et al. 2018 Bifeld et al. This content is distributed under the terms of the Creative Commons Attribution 4.0 International license.

10.1128/mSystems.00214-18.4 TABLE S2 RiboTaper analysis of ribosome profiling and RNA-Seq reads of L. donovani WT-RAD. Listed are gene ID, product description, coding sequence length (nt), number of P-sites per CDS, P-sites per CDS adjusted by total reads per sample, P-sites per CDS adjusted by median reads per CDS of sample, RNA sites per CDS, RNA sites per CDS adjusted by total reads per sample, RNA sites per CDS adjusted by median reads per CDS of sample, translation efficiency, and log_2_ of TE. P-sites, protected sites; CDS, coding sequence; adjusted P-sites, P-sites/million reads per sample; RNA sites/CDS, number of aligned RNA-Seq sites per coding sequence; adjusted RNA sites/CDS, number of aligned RNA sequence reads per coding sequence/million reads per sample; TE, translation efficiency = P-sites/RNA sites. Download Table S2, XLS file, 1.9 MB. Copyright © 2018 Bifeld et al. 2018 Bifeld et al. This content is distributed under the terms of the Creative Commons Attribution 4.0 International license.

10.1128/mSystems.00214-18.5 TABLE S3 RiboTaper analysis of ribosome profiling and RNA-Seq reads of L. donovani WT+RAD. Analysis as described for Table S2. Download Table S3, XLS file, 1.9 MB. Copyright © 2018 Bifeld et al. 2018 Bifeld et al. This content is distributed under the terms of the Creative Commons Attribution 4.0 International license.

10.1128/mSystems.00214-18.6 TABLE S4 RiboTaper analysis of ribosome profiling and RNA-Seq reads of L. donovani Hsp90rr+RAD. Analysis as described for Table S2. Download Table S4, XLS file, 1.8 MB. Copyright © 2018 Bifeld et al. 2018 Bifeld et al. This content is distributed under the terms of the Creative Commons Attribution 4.0 International license.

**FIG 2:**
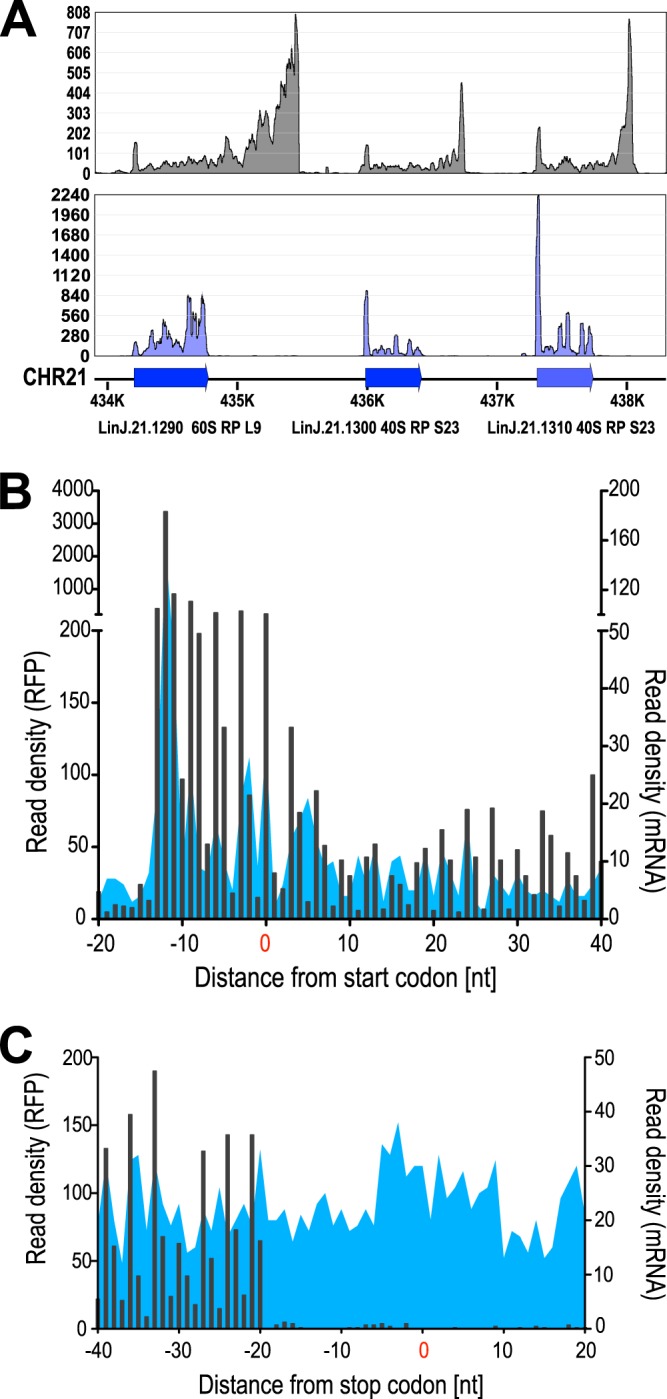
Verification of ribosome footprints. (A) Read mapping analysis of RNA-Seq (gray) and RFPs (purple) on a cluster of three ribosomal protein-coding genes on chromosome 21. Blue arrows delineate annotated coding sequences with gene IDs. Read alignment densities for RNA-Seq (top panel) and RFP (bottom panel) are depicted. (B and C) The 30-nt ribosome footprints (black bars) of the representative sample HSP90rr+RAD were mapped to the annotated CDSs in relation to the initiation (B) and termination (C) sites and plotted by the first nucleotides of the P-sites. Corresponding RNA-Seq reads are shown as blue peaks. Note that peak densities occur at 3-nt intervals for RFPs, representing 3-nt increments of ribosome movement.

Figure 2A shows RFPs (purple) aligning accurately to three annotated CDSs for ribosomal proteins on chromosome 21 while the coverage by RNA-Seq reads (gray) extends into the putative UTRs. Furthermore, the RFPs show the expected 3-bp periodicity (Fig. 2B and C), reflecting the 3-nt increments of ribosome translocation during translation and thus supporting the notion that the RNase-protected fragments are indeed the result of a ribosome footprint. Such periodicity is not observed with the transcriptome-derived reads (Fig. 2B and C).

**FIG 3:**
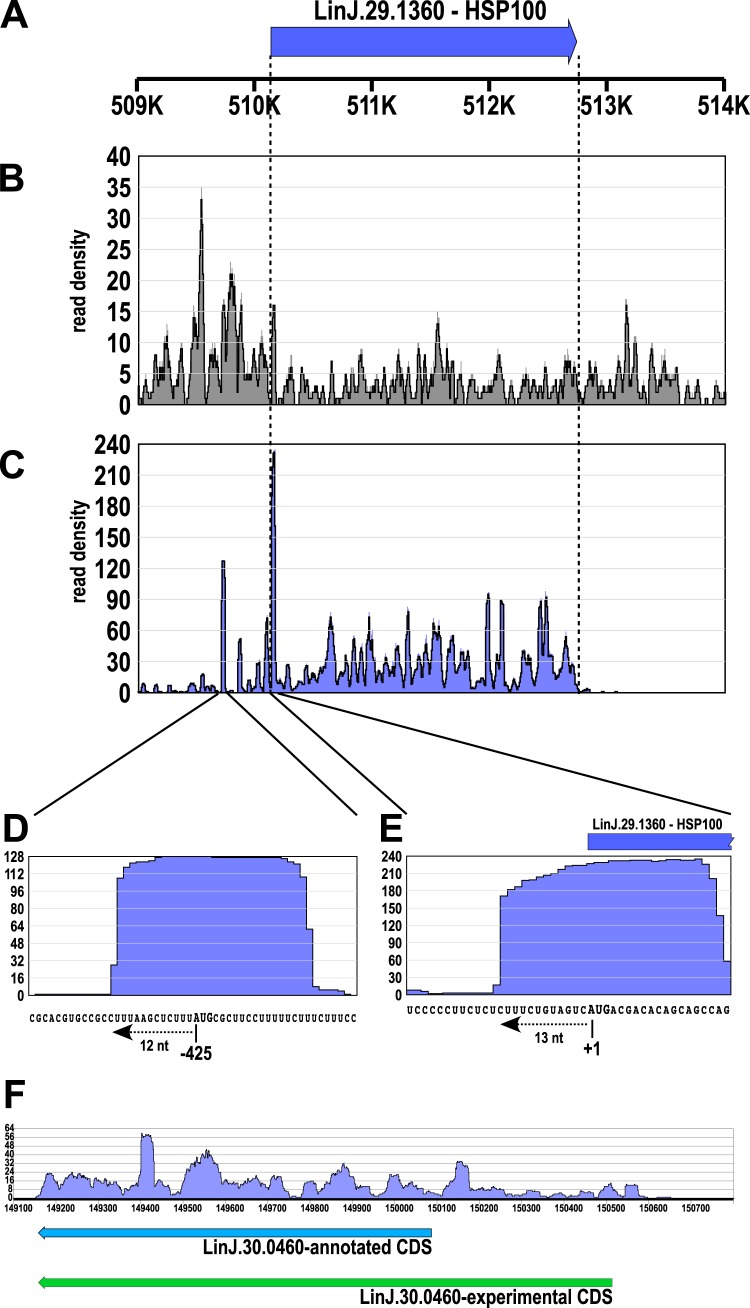
Identification of uORFs and extended coding sequences. (A) Position of the HSP100 CDS on chromosome 29; ruler shows position in kilobase pairs. (B) RNA-Seq read alignment at the HSP100 gene locus. (C) RFP read alignment at the HSP100 gene locus. (D) Enlargement of the 5′ UTR of HSP100 showing RFPs aligned to an upstream AUG codon, representing a putative uORF. (E) Zoom into the translation initiation site of HSP100 represented by RFP reads aligning 12 nucleotides upstream of the AUG start codon. The blue bar represents the start of the HSP100 CDS. (F) Possible erroneous annotation of the LinJ.30.0460 (eIF4-E4) CDS, with RFP reads aligning well upstream of the annotated AUG start codon. Annotated and experimentally determined CDSs are shown as arrows.

Several short upstream open reading frames (uORFs) in the 5′ UTRs of genes (55–57) could also be identified in *Leishmania* by visual inspection of RFP read patterns. A systematic search was not possible due to the lack of UTR data in the available genome annotation. One interesting example is the gene for heat shock protein 100 (HSP100) on chromosome 29 (LinJ29.1360, Fig. 3A). While the transcriptome-derived reads cover the 5′ and 3′ untranslated regions (UTRs) (Fig. 3B) that were determined previously (33, 58), RFPs map to the annotated ORF (Fig. 3C) starting 13 nt upstream of the predicted start codon (Fig. 3E). In addition, RFP reads also show alignment to a 30-bp region found 425 nt upstream of the HSP100 start codon. This ∼30-bp footprint starts 12 nt upstream of an AUG codon (Fig. 3D), with an in-frame termination codon located 45 nt downstream, and can thus be considered a uORF, possibly regulating heat shock-induced translation of HSP100 (31, 33). The putative uORF is in a different reading frame than the HSP100-coding sequence.

We also observed instances of false gene annotation, e.g., the gene for translation initiation factor 4E-4 (LinJ.30.0460) is annotated to the region from position 150087 to 149161 on chromosome 30, encoding a 308-aa polypeptide. Our data (Fig. 3F) suggest that LinJ.30.0460 translation initiates 420 nucleotides upstream, at position 150507, and rather encodes a 448-aa protein. Indeed, this is confirmed by the annotation of the L. donovani BPK282A1 genome. Freire et al. also assumed a missing N terminus in the annotation of LmjEIF4E4 after sequence comparison with the T. brucei orthologue (59).

From our data we conclude that the RFPs faithfully represent the translatome of L. donovani.

### Gene regulation in *Leishmania*.

We next calculated the changes of ribosome footprinting (RFP) density, RNA abundance (RNA), and translation efficiency (TE) induced by challenge with radicicol (RAD) in the presence or absence of RAD-resistant HSP90rr, using the data in Table S2, Table S3, and Table S4. We used the RiboTaper analysis tool to obtain the RFP and RNA read densities for all 8,240 genes. Of these, 283 genes were eliminated due to lack of aligned reads in any of the 6 data sets (not shown). RFP and RNA read counts were normalized using the median counts per CDS for each sample (Table S1). RFP and RNA counts were then log_2_-transformed.

**FIG 4:**
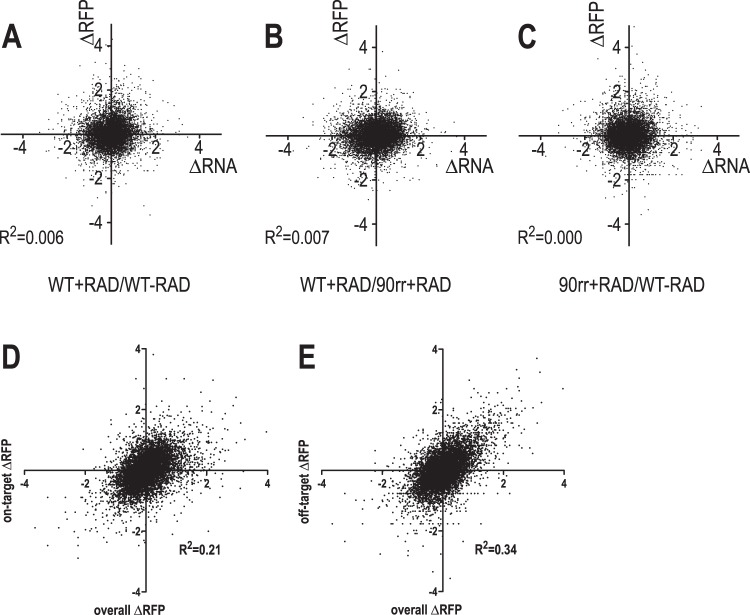
Correlation analysis. ΔRFP was plotted against ΔRNA for (A) WT+RAD/WT-RAD, (B) WT+RAD/HSP90rr+RAD, and (C) HSP90rr+RAD/WT-RAD. The coefficients of determination, *R*^2^, are displayed. (D) Plot of overall ΔRFP (WT+RAD/WT-RAD) against on-target ΔRFP (WT+RAD/HSP90rr+RAD) with coefficient of determination, *R*^2^. (E) Plot of overall ΔRFP (WT+RAD/WT-RAD) against off-target ΔRFP (HSP90rr+RAD/WT-RAD) with coefficient of determination, *R*^2^.

We then determined the coefficient of determination (*R*^2^) between the ΔRFP (variation of ribosome footprinting densities) and the ΔRNA (variation of RNA abundance). This was performed for WT+RAD versus WT-RAD (overall RAD effects), WT+RAD versus HSP90rr+RAD (on-target RAD effects), and HSP90rr+RAD versus WT-RAD (off-target RAD effects) and for 7,957 genes (Fig. 4A to C; Table S5). *R*^2^ values for ΔRNA versus ΔRFP are close to zero, meaning that the RNA abundance does not determine protein synthesis rates as measured by ribosome footprinting. We conclude that RAD-induced gene expression changes must be regulated at the translation level.

10.1128/mSystems.00214-18.7 TABLE S5 Pairwise comparisons of WT+RAD versus WT-RAD, WT+RAD versus Hsp90rr+RAD, and Hsp90rr+RAD versus WT-RAD. The ΔRFP (ribosome footprints), ΔRNA, and ΔTE are given for each gene and comparison as log_2_ values. Listing by gene ID. Data rows containing values of 0 (zero) were excluded. Download Table S5, XLS file, 1.8 MB. Copyright © 2018 Bifeld et al. 2018 Bifeld et al. This content is distributed under the terms of the Creative Commons Attribution 4.0 International license.

We next compared the overall ΔRFP values (WT+RAD versus WT-RAD) with either the on-target ΔRFP (WT+RAD versus HSP90rr+RAD, Fig. 4D) or the off-target ΔRFP (HSP90rr+RAD versus WT-RAD, Fig. 4E) and determined the coefficient of determination, *R*^2^. Both plots suggested an intermediate degree of correlation of *R*^2^ = 0.21 and *R*^2^ = 0.34, respectively. This indicates that overall RAD effects on protein synthesis are more likely due to off-target inhibition by RAD than to HSP90-specific, on-target activity, raising the question on which targets other than HSP90 (HSP83) RAD may be active.

### RAD-induced protein synthesis changes.

Using the same RiboTaper analysis data (Table S5), we next identified proteins that are induced or reduced >2-fold under RAD treatment (Table S6), and from these we grouped proteins by function (Table 1; Table S7).

**TABLE 1:** List of induced proteins by functional groups^*a*^

Category and gene ID	Annotation	WT+RAD vs WT-RAD		
		ΔRFP	ΔRNA	ΔTE
Protein folding/chaperones				
LinJ.22.0670	A2 protein	+2.873	+0.576	+2.297
LinJ.33.0940	DnaJ, putative	+1.169	−0.241	+1.410
LinJ.27.2350	DnaJ, putative	+1.059	+0.550	+0.509
LinJ.25.2290	DnaJ/zinc-finger double-stranded RNA-binding, putative	+1.064	+0.813	+0.250
LinJ.29.1360	HSP100	+0.674	+0.301	+0.373
LinJ.34.0230	HSP23	+1.086	−0.495	+1.581
LinJ.28.3040	HSP70, putative	+1.024	−0.328	+1.352
LinJ.33.0350	HSP90	+1.601	+0.372	+1.230
LinJ.30.2480	mtHSP70	+1.535	+0.321	+1.215
LinJ.04.0710	Tir chaperone protein (CesT) family	−1.076	+0.431	−1.507
LinJ.36.2190	TPR repeat, putative	+1.027	+0.607	+0.420
LinJ.26.0360	TPR repeat, putative	−1.078	+0.000	−1.078
LinJ.05.0410	TPR repeat, putative	−1.160	−0.677	−0.483
Redox enzymes				
LinJ.32.2880	As/Sb reductase, putative	+1.256	−0.919	+2.175
LinJ.34.0070	Ascorbate peroxidase, putative	+1.845	+0.481	+1.363
LinJ.31.2600	Ferredoxin, 2Fe-2S-like protein	+0.934	+0.122	+0.813
LinJ.27.0670	Glutaredoxin-like protein	+1.396	−0.241	+1.637
LinJ.26.0770	Glutathione peroxidase-like protein, putative	+2.512	+0.576	+1.936
LinJ.26.0780	Glutathione peroxidase-like protein, putative	+1.274	+0.582	+0.692
LinJ.32.1910	Iron superoxide dismutase, putative	+1.275	+0.010	+1.265
LinJ.23.0500	Trypanothione synthetase, putative	+1.207	−0.060	+1.268
LinJ.29.1250	Tryparedoxin 1	+1.170	+0.430	+0.740
LinJ.15.1140	Tryparedoxin peroxidase	+1.120	+0.235	+0.885
LinJ.15.1100	Tryparedoxin peroxidase	+1.029	+0.406	+0.623
Proteolytic enzymes				
LinJ.26.2720	CAAX prenyl protease 2, putative	+1.270	+0.122	+1.148
LinJ.14.0920	Calpain-like cysteine peptidase, putative	+2.157	+0.247	+1.910
LinJ.20.1210	Calpain-like cysteine peptidase, putative	+1.067	+0.045	+1.023
LinJ.36.6520	Carboxypeptidase, putative	+1.700	+0.177	+1.523
LinJ.14.0180	Carboxypeptidase, putative	+1.051	+0.339	+0.712
LinJ.22.1540	Metallopeptidase, clan MA(E), family M3, putative, partial	+1.746	−0.475	+2.222
LinJ.36.4230	Metallopeptidase, clan MC, family M14, putative	+1.022	+0.268	+0.754
LinJ.09.1360	PPPDE putative peptidase domain-containing protein, putative	+1.369	−0.530	+1.900
LinJ.34.4390	Proteasome beta 7 subunit, putative	+2.134	−0.610	+2.745
LinJ.01.0730	Ubiquitin-activating enzyme, putative	+1.050	−0.129	+1.179
LinJ.28.0500	Ubiquitin-activating enzyme, putative	−1.348	−1.345	−0.003
LinJ.36.4580	Ubiquitin protein ligase, putative (fragment)	+1.256	−0.134	+1.390
LinJ.32.0730	Ubiquitin-conjugating enzyme E2, putative	+1.099	+0.759	+0.340
LinJ.21.0500	Ubiquitin-conjugating enzyme-like protein	+1.026	+0.278	+0.747
LinJ.31.1930	Ubiquitin-fusion protein	+2.716	+0.889	+1.826
LinJ.13.0620	Ubiquitin-like protein	+1.185	+0.145	+1.040
Amastin family				
LinJ.30.1490	Ama1 protein, putative	+1.256	−0.521	+1.777
LinJ.08.0650	Amastin surface glycoprotein, putative	+1.058	+0.421	+0.637
LinJ.08.0780	Amastin-like protein	+2.157	+1.218	+0.938
LinJ.08.0680	Amastin-like protein	+2.104	+0.122	+1.983
LinJ.34.1040	Amastin-like protein	+2.078	+0.374	+1.704
LinJ.29.1450	Amastin-like protein	+1.513	+0.806	+0.706
LinJ.08.0760	Amastin-like protein	+1.034	+0.759	+0.275
LinJ.24.1300	Amastin-like surface protein-like protein	+2.597	−0.589	+3.186
LinJ.34.1680	Amastin-like surface protein, putative	+2.303	−0.241	+2.544
LinJ.34.1010	Amastin-like surface protein, putative	+1.809	+0.566	+1.242
LinJ.34.1020	Amastin-like surface protein, putative	+1.527	+0.264	+1.263
LinJ.34.1690	Amastin-like surface protein, putative	+1.349	−0.978	+2.327
LinJ.34.1730	Amastin-like surface protein, putative	+1.031	+0.185	+0.845
LinJ.34.1150	Amastin-like surface protein, putative	+1.015	+0.396	+0.619
LinJ.29.3010	Amastin, putative	+4.303	+0.049	+4.255
LinJ.29.3030	Amastin, putative	+2.283	+0.555	+1.727
LinJ.31.0460	Amastin, putative	+1.443	+0.213	+1.230
LinJ.29.3000	Amastin, putative	+1.157	+0.174	+0.983
Chromatin proteins				
LinJ.20.0460	Cell cycle checkpoint protein RAD1-like, putative (fragment)	−1.481	−0.978	−0.503
LinJ.28.2550	DNA replication licensing factor MCM6, putative	−1.098	−1.148	+0.050
LinJ.09.0930	Histone H1-like protein	+1.041	+0.280	+0.761
LinJ.09.0930	Histone H1-like protein	+1.041	+0.280	+0.761
LinJ.27.1120	Histone H1, putative	+1.387	+0.377	+1.011
LinJ.27.1070	Histone H1, putative	+1.040	+0.096	+0.944
LinJ.27.1120	Histone H1, putative	+1.387	+0.377	+1.011
LinJ.27.1070	Histone H1, putative	+1.040	+0.096	+0.944
LinJ.29.1850	Histone H2A, putative	+5.007	+1.081	+3.927
LinJ.29.1870	Histone H2A, putative	+2.050	−0.759	+2.809
LinJ.29.1850	Histone H2A, putative	+5.007	+1.081	+3.927
LinJ.29.1870	Histone H2A, putative	+2.050	−0.759	+2.809
LinJ.19.0040	Histone H2B	+2.688	−0.796	+3.484
LinJ.09.1410	Histone H2B	+1.532	−0.493	+2.025
LinJ.19.0040	Histone H2B	+2.688	−0.796	+3.484
LinJ.09.1410	Histone H2B	+1.532	−0.493	+2.025
LinJ.16.0600	Histone H3, putative	+1.240	−0.505	+1.744
LinJ.16.0600	Histone H3, putative	+1.240	−0.505	+1.744
LinJ.36.0020	Histone H4	+1.443	−1.328	+2.771
LinJ.15.0010	Histone H4	+1.034	−0.210	+1.245
LinJ.35.0020	Histone H4, putative, pseudogene	+1.204	−0.108	+1.313
LinJ.30.1010	Histone-binding protein RBBP4, putative	−1.388	−1.463	+0.076
LinJ.26.0710	Regulator of chromosome condensation (RCC1) repeat, putative	+1.671	−0.189	+1.860
Protein kinases				
LinJ.27.1680	Casein kinase I-like protein	−2.651	+0.022	−2.673
LinJ.29.2260	Cdc2-related kinase 10, putative	+1.231	+0.039	+1.191
LinJ.33.1930	Dual-specificity protein kinase, putative	+1.993	+0.929	+1.064
LinJ.35.4060	Protein kinase A catalytic subunit isoform 1	+1.335	+0.185	+1.149
LinJ.32.1350	Protein kinase domain-containing protein, putative	+1.619	+0.039	+1.580
LinJ.32.1350	Protein kinase domain-containing protein, putative	+1.619	+0.039	+1.580
LinJ.29.0380	Protein kinase-like protein	+1.267	+0.921	+0.346
LinJ.17.0440	Protein kinase, putative	+2.207	+0.451	+1.756
LinJ.35.4690	Protein kinase, putative	+1.256	−1.700	+2.957
LinJ.14.1510	Protein kinase, putative	+1.050	+0.633	+0.416
LinJ.19.0590	Protein kinase, putative	−1.191	−0.300	−0.891
LinJ.19.1510	Protein kinase, putative	−1.329	+0.344	−1.673
LinJ.19.1640	Protein kinase, putative	−1.651	−1.241	−0.410
LinJ.28.3240	Serine/threonine kinase, putative	+1.157	−0.978	+2.135
Fatty acid metabolism				
LinJ.14.0770	Fatty acid elongase, putative	+1.934	−0.826	+2.760
LinJ.14.0710	Fatty acid elongase, putative	+1.083	−0.027	+1.109
LinJ.14.0720	Fatty acid elongase, putative	−1.124	+0.344	−1.468
LinJ.14.0750	Fatty acid elongase, putative	−1.236	−0.241	−0.995
LinJ.14.0670	Fatty acid elongase, putative	−1.622	+0.149	−1.771
LinJ.01.0520	Fatty acyl-CoA synthetase 2, putative	−1.918	−0.794	−1.124
LinJ.03.0220	Long-chain fatty acyl-CoA synthetase, putative	−1.482	−0.241	−1.241
LinJ.01.0540	Long-chain-fatty acid-CoA ligase, putative	−1.663	−0.014	−1.650

a Positive numbers indicate log_2_ increases, while negative numbers indicate log_2_ decreases.

10.1128/mSystems.00214-18.8 TABLE S6 Pairwise comparisons of WT+RAD versus WT-RAD, WT+RAD versus Hsp90rr+RAD, and Hsp90rr+RAD versus WT-RAD, filtered for ΔRFP values >1 and <−1. Columns C, D, and E give information of known association with cellular compartments, gene function, and the cellular processes. Download Table S6, XLS file, 0.2 MB. Copyright © 2018 Bifeld et al. 2018 Bifeld et al. This content is distributed under the terms of the Creative Commons Attribution 4.0 International license.

10.1128/mSystems.00214-18.9 TABLE S7 Pairwise comparisons of WT+RAD versus WT-RAD, WT+RAD versus Hsp90rr+RAD, and Hsp90rr+RAD versus WT-RAD, by functional groups of proteins. Download Table S7, XLS file, 0.04 MB. Copyright © 2018 Bifeld et al. 2018 Bifeld et al. This content is distributed under the terms of the Creative Commons Attribution 4.0 International license.

We first looked at protein folding catalysators, i.e., chaperones and other heat shock proteins. Surprisingly, only a few heat shock proteins, i.e., HSP90, HSP70, mtHSP70, and HSP23, show induced synthesis under heat stress. This induction is not abrogated by overexpression of the RAD-resistant HSP90rr, indicating an induction via off-target RAD effects. The A2 protein, a stress protein correlated with promastigote-to-amastigote differentiation (60), shows the strongest induction, in keeping with the observed pro-amastigote effect of RAD (14, 15). This induction appears to be mediated by inactivation of HSP90 since the effect is abrogated by HSP90rr expression.

RAD treatment also induces a number of redox enzymes implicated in the oxidative stress response, among them members of the glutathione and trypanothione pathways. This is in keeping with the need of amastigotes to adapt to the oxidative environment of macrophage lysosomes. RAD treatment also upregulates the synthesis of proteolytic enzymes, such as various peptidases and ubiquitin tagging pathways, reflecting the need for proteolytic degradation during the cellular differentiation from promastigotes to amastigotes.

Surface proteins of the amastin family are known surface markers of the amastigote stage (61). Our analysis shows 18 amastin family members with a more-than-2-fold increase of synthesis under RAD treatment, with the majority activated through off-target RAD effects. This further supports the correlation between RAD treatment and differentiation toward the amastigote stage.

The synthesis of most histones is increased under RAD treatment by a combination of HSP90-specific and off-target RAD effects. This indicates an increased need for nucleosomal packing of DNA under RAD challenge, either mimicking a feature of amastigotes or reflecting the growth arrest caused by HSP90 inhibition (14, 15).

The effect of RAD on ribosomal protein and translation factor synthesis is ambiguous, with roughly the same number of proteins showing increased or reduced synthesis. This may reflect the observed reprogramming of translation during RAD treatment.

We also observe mostly increased synthesis of several known or putative protein kinases. None of the kinases we found affected by RAD treatment has been ascribed a role in the stress response or in stage conversion.

A functional group of proteins that are largely negatively affected by RAD treatment comprises enzymes of the fatty acid synthesis, possibly reflecting a reported shift to fatty acid catabolism for amastigotes (21). This is further underscored by a gene ID-based metabolic pathway analysis (not shown) which also identifies fatty acid metabolic pathways as negatively affected. This may reflect the metabolic changes during promastigote-to-amastigote differentiation. These negative effects are mostly due to HSP90-specific RAD effects.

## DISCUSSION

In most eukaryota and prokaryota, the expression of specific genes or operons is controlled at the level of RNA synthesis and RNA processing, allowing an approximate quantitative assessment of gene expression patterns by analyzing the steady-state level of gene-specific mRNAs. However, control of gene expression is also exerted independently of RNA abundance, at the levels of translation initiation and elongation (62). This is even more important in *Leishmania* spp., where no gene-specific transcription regulation exists (25) and where the correlation of transcriptome and proteome is poor (32, 63). In contrast, translatome data—obtained by ribosome profiling—correlate well with proteome data (45, 48), even reflecting the subunit stoichiometry of multiprotein complexes (64). Thus, ribosome profiling provides a reliable option for the quantification of translation across the transcriptome, which is especially useful for trypanosomatids as these parasites rely on posttranscriptional control of gene expression (65).

Our ribosome profiling analysis indeed provides a representative view of L. donovani gene expression. An average of 33% of the qualified reads aligned to annotated open reading frames (see Table S1 in the supplemental material), a value that compares well with the earlier reported 16% for Saccharomyces cerevisiae (45). We also found >97% of the annotated CDS in the L. donovani genome represented by the RFP reads (not shown). RNA-Seq reads show a similarly high coverage at 99%, with 30% of the qualified reads mapping to CDS. This lower value reflects the absence of untranslated regions (UTRs) from the database we used for alignment.

Ribosomes are macromolecules with the A, P, and E decoding sites (66), resulting in a 3-nt periodicity of movement along the mRNA molecule. This is reflected in our results by the observed 3-nt periodicity of the RFP read alignments (Fig. 2B and C). Because the ribosome occupies 26 to 30 bases on the mRNA molecule, with an AUG or a stop codon in the P-site, the coverage with RFP reads should start approximately 12 nucleotides upstream of the start codon (AUG) and end ∼18 nucleotides upstream of the stop codon, which is indeed reflected in our results (Fig. 2B and C). Thus, the coverage by RFP reads displays the actively translated mRNA sequences in L. donovani. Moreover, the unusual genome organization of trypanosomatids may lead to incorrect annotations by using standard algorithms for the ORF identification, which may be corrected by RFP densities. This was shown for T. brucei (50) and for L. donovani, e.g., for the gene LinJ.30.0460 (Fig. 3F).

Due to their peculiar transcription and maturation of mRNA, trypanosomatids rely on posttranscriptional regulation for their adaptation to environmental changes. Posttranscriptional regulation in eukaryotes often depends on *cis*-acting elements in the mRNA 5′ UTR, such as internal ribosomal entry sites (IRESs), which allow a cap-independent translation initiation (56, 67), or short open reading frames upstream of the protein-coding sequence (uORFs), which have regulatory capacities (55–57). In trypanosomatids, the translation regulation is commonly accepted as being directed by elements located in the 3′ UTR (65, 68). However, the 5′ UTR of the L. mexicana HSP83 (=HSP90) gene was shown to be crucial for the translation initiation of the CDS (68), demonstrating the regulatory function of an as-yet-unidentified *cis*-acting element. Moreover, uORFs were found in L. mexicana mRNAs by transcriptome analysis (69) as well as in T. brucei by applying ribosome profiling (49, 50). We applied RiboTaper (54) to our ribosome profiling-derived raw data, which identified annotated CDSs but no uORFs (not shown), due to the lack of UTR sequences in the available genome annotation. However, by manual inspection of the RFP read densities on genes subject to stage-specific expression control, we were able to identify at least one uORF located upstream of the HSP100 gene (LinJ.29.1360) (Fig. 3C), which starts 12 nt upstream of an initiation codon and contains an in-frame termination codon (Fig. 3D). uORFs initiate with either an AUG or a non-AUG start codon, terminate with in-frame stop codons (70–72), and are considered translation-reducing elements, as they capture some of the scanning preinitiation complexes (55, 57). However, uORFs also promote translation of particular mRNAs under cell stress conditions (57, 70, 73), and non-AUG uORFs are found in the 5′ UTRs of a variety of chaperones (74). Thus, the identified ribosome-protected sequence upstream of the HSP100 ORF (Fig. 3D) is likely an uORF and may serve as a regulatory element for translation in L. donovani. In keeping with this hypothesis, HSP100 expression is indeed temperature induced (33).

For the longest time, the *Leishmania* HSP90 was inaccessible to genetic analysis due to its high number of identical, tandemly repeated gene copies (27, 36) and the essential nature of this major chaperone. The availability of the HSP90-specific inhibitors geldanamycin and RAD allowed a first assessment of the importance of HSP90 for the parasites’ life cycle control and their stage-specific gene expression (14, 75). This was then augmented by the use of an inhibitor-resistant, phenotypically neutral variant of HSP90, HSP90rr (15), which allows monitoring of the phenotypic effects of point mutants in a conditional setting. This also confirmed that the effects of the HSP90 inhibitor RAD on the morphology were due to the RAD-HSP90 interaction. In this context, dosage is of critical importance. High concentrations of geldanamycin cause a growth arrest in the G_2_ cell cycle phase (14). HSP90 inhibitors, e.g., the antitumor drug candidate 17-AAG, a derivative of geldanamycin, may even find a use as antileishmanial therapeutics, having activity against cutanotropic leishmaniae both *in vitro* (76) and *in vivo* (77). Those findings are in keeping with our observation that intracellular leishmaniae depend on HSP90 function (15).

Earlier work (14, 15) indicated that the majority of the effects of geldanamycin and RAD can be attributed to HSP90 inhibition. In the light of our findings, this view must be reconsidered. The availability of the RAD-resistant HSP90rr transgene allowed us to differentiate between target-specific effects due to HSP90 inhibition which were absent under ectopic HSP90rr expression and effects of RAD in the HSP90rr-expressing cells that were absent from the untreated WT-RAD samples and that we consider off-target. This result was unexpected since previous work showed that ectopic HSP90rr expression reverted all phenotypic effects of RAD treatment in promastigotes and intracellular amastigotes (15).

Since RAD interacts specifically with the nucleotide binding sites of HSP90 chaperone family members, but not with those of other ATP-hydrolyzing chaperones (78), we suspect that the off-target interactions of RAD inhibition in *Leishmania* are with two other HSP90 paralogues, namely, GRP94/LPG3 (40) and/or HSP75/TRAP-1 (41). While the former has been described as essential for L. donovani lipophosphoglycan synthesis (40, 79), the latter is part of the protein payload of immunomodulatory *Leishmania* exosomes and dependent on HSP100 for its exosomal localization (42). While HSP90 synthesis is increased via HSP90 inhibition, neither GRP94/LPG3 nor TRAP-1/HSP75 is induced under RAD inhibition (Table S5). Both GRP94 and TRAP-1 chaperones are known to bind RAD with HSP90-like affinity (78, 80, 81).

**FIG 5:**
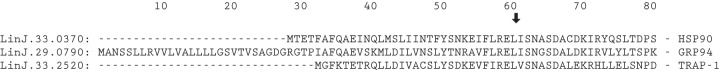
Sequence alignment of HSP90 chaperone family members in L. infantum. MUSCLE alignment of the deduced N-terminal amino acid sequences of 3 HSP90 paralogues, with the conserved Leu residues of HSP90 indicated by an arrow.

The Leu_33_ residue of HSP90 is conserved in GRP94 and in TRAP-1 (Fig. 5). It may be interesting to express variants of GRP94 and TRAP-1 with an equivalent Leu_60_Ile and Leu_29_Ile exchange, respectively, to see which of them may counteract the off-target (OT) effects of RAD. Such knowledge would be important, since several proteins of importance are controlled via the pathway(s) affected by the OT effects. Nevertheless, RAD may also target another protein(s) in *Leishmania* that has so far escaped identification as potential targets.

It is noteworthy that GRP94/lpg3 null mutants are viable *in vitro* but entirely lack synthesis of lipophosphoglycans (40), important surface molecules that promote *Leishmania* survival early in the infection. It will therefore be interesting to see whether the negative effects of RAD on the *in vitro* infectivity (15) may be due to inactivation of GRP94.

RAD treatment causes changes in the synthesis of several groups of proteins. It was shown before that the abundance of heat shock proteins HSP90, HSP70, and HSP100 and also of the amastigote-specific A2 proteins increases under supposed HSP90 inhibition (14). Indeed, synthesis of these proteins increases between 1.6-fold and 7.5-fold under RAD treatment. Other notable heat shock proteins in this group are HSP23, a major facilitator of thermotolerance in *Leishmania* (34), and the mitochondrial HSP70. The other examples in this group are putative chaperones due to structural features.

The synthesis of 12 redox proteins is also induced under RAD, three by target-specific regulation and three by off-target effects, with the rest showing a mixture of both. The proteins of this group belong to the oxidative stress protection pathways of the parasites that facilitate the survival of amastigotes in the host macrophages. For instance, ascorbate peroxidase was shown to protect *Leishmania* against oxidative stress-induced apoptosis (82). Trypanothione synthase and trypanothione have been linked to antimony resistance and viability (83, 84).

Under conditions of cell stress, damaged proteins must be recognized and directed to proteolytic degradation in the proteasome. This is facilitated by binding to heat shock proteins and conjugation with ubiquitin. Seven members of the ubiquitin pathway and one proteasome subunit are induced under RAD-simulated cell stress. Moreover, a number of peptidases are also synthesized at higher rates, possibly reflecting the need for proteolytic activity during the size reduction of the parasite when it converts from the longish promastigote to the ovoid amastigote, one of the phenotypic effects of RAD.

No fewer than 18 members of the amastin surface proteins are found to be synthesized at increased rates. Amastins are a large family of transmembrane surface proteins, expressed predominantly in the amastigote stage (28, 61), that are linked to Leishmania donovani tropism (85) and intracellular survival of L. braziliensis (86). Their upregulation, mostly by presumed off-target activity of RAD, is further indication that RAD treatment activates amastigote-specific gene expression beyond heat shock and other stress proteins.

The upregulation of multiple histone proteins under RAD indicates a higher nucleosome density under RAD. However, a detailed proteome study showed no evidence of increased histone protein abundance in *in vitro*-differentiated amastigotes (21).

Inhibition of HSP90 also has a negative impact on the fatty acid synthesis, in keeping with the changes of metabolic pathways observed by proteome analysis of axenically cultivated amastigotes (21).

The inhibition of HSP90 triggers multiple changes in the gene expression of *Leishmania*, suggesting an inhibitory effect of this major chaperone on the expression of several stress-induced genes. In parallel, RAD also appears to have an effect on the protein synthesis patterns via off-target interactions, possibly with other HSP90 family chaperones. Still, a natural modulation of HSP90 activity may be one way leading to life cycle stage-specific gene expression in a protozoan that completely lacks control of individual gene transcription and that regulates gene expression independently of mRNA steady-state levels (32; this paper). Such natural modulation pathways may include protein kinases, since it was shown that HSP90 and several associated chaperones and cochaperones are the subjects of amastigote stage-specific protein phosphorylation (87). The recent finding that HSP90 and HSP70 are both substrates for MAP kinase 1 (88) supports this idea, since MAP kinase 1 or LmxMPK1 is crucial for the intracellular survival of *Leishmania* (89). Another kinase recently shown (A. Hombach-Barrigah, K. Bartsch, D. Smirlis, H. Rosenqvist, A. MacDonald, F. Dingli, D. Loew, G. F. Späth, N. Rachidi, M. Wiese, and J. Clos, unpublished data) to catalyze HSP90 phosphorylation is casein kinase 1.2 (90, 91), which is crucial for promastigote growth (92) and is also found in the HSP90-containing exosome-like vesicles that are shed by *Leishmania* as a means for host cell immune modulation (42, 93). Neither kinase is upregulated upon HSP90 inhibition (Table S5). One may speculate that stage-specific roles played by protein kinases are transduced through reversible modulation of HSP90 activity. Once protein kinases and their target sites on HSP90 are unraveled, the availability of HSP90 phosphorylation site mutations (A. Hombach, unpublished data) combined with the power of ribosome profiling analysis can be expected to test this hypothesis.

## MATERIALS AND METHODS

### *Leishmania* cell culture.

Promastigote L. donovani strain 1SR (MHOM/SD/62/1SR) was cultured at 25°C in growth medium based on Medium 199 (Sigma-Aldrich) supplemented with 25% heat-inactivated FCS, 40 mM HEPES, pH 7.4, 0.2% NaHCO_3_, 100 µM adenine, 1.2 µg ml^−1^ 6-biopterin, 10 µg ml^−1^ heme, and 1× Pen/Strep/l-glutamine (Sigma), pH 7.0. Strain L. donovani [HSP90rr] (15) was maintained under G418 selection (100 µg/ml) until 24 h before exposure to radicicol.

### *Leishmania* cell harvest and lysis.

The parasites were counted using a CASY cell counter (Roche) and added at a cell density of 4 × 10^6^ ml^−1^ into 150 ml of growth medium containing 5 ng ml^−1^ RAD (Sigma). The parasites were allowed to proliferate for 72 h at 25°C. The further protocol was adapted from reference 52. Briefly, cycloheximide (Sigma) was added to the parasite cultures at a final concentration of 100 µg ml^−1^ and incubated for 5 min at room temperature. Three biological samples with an average cell count of 1 × 10^9^ each were collected by centrifugation at 3,000 × *g* and 4°C for 5 min, washed once by resuspension in 1 ml polysome lysis buffer (15 mM Tris-HCl, pH 7.4, 0.3 M KCl, 5 mM MgCl_2_, 0.5 mM DTT, 100 µg ml^−1^ cycloheximide, and 1 mg ml^−1^ heparin), and pelleted at 3,000 × *g* and 4°C for 5 min. The supernatant was discarded. The cells were transferred to 1.5-ml microcentrifuge tubes using 1 ml of ice-cold polysome lysis buffer and pelleted again at 10,000 × *g* and 4°C for 40 s. The cells were lysed by adding 400 µl polysome lysis buffer containing 1% Triton X-100 and 10 units of Turbo DNase I (Ambion) followed by incubation on ice for 30 min. The RNA concentrations of the cell lysates was determined at OD_260_ using a NanoDrop 2000 (Thermo Fisher Scientific).

### Preparation of the sequencing libraries and footprinting of RNA and mRNA.

The sequencing libraries were prepared as described previously (50).

Briefly, for the purification of ribosome footprint RNAs, the polysomes in the cell lysates were disjoined to monosomes by digesting away unprotected RNA. Aliquots of 200 µl (OD_260_ = 50) were treated with 1,600 U of RNase I (Ambion) and incubated on ice for 1 h. The RNA digestion was stopped by adding 100 U of RNasin RNase inhibitor (Promega) to the aliquots. For the undigested control, 100 U of RNasin RNase inhibitor was added to RNase I-untreated aliquots of each sample. The RNase I-treated samples were loaded onto a sucrose gradient (10% [wt/vol] to 50% [wt/vol]) as described in Ingolia et al. (45). Gradients were fractionated on the Gradient Station (Science Services) based on their absorbance at *A*_254_. Fractions representing the monosome peak were collected at 50-mm distance starting at 0.05 *A*_254_ absorbance.

Footprint RNA from the monosome fraction and total RNA from the undigested cell fraction were purified by a hot (65°C) acid phenol-chloroform-isoamyl alcohol (vol/vol/vol, 25:24:1) extraction as described by Ingolia (94).

The mRNA isolation from the total RNA fraction and the following mRNA fragmentation were performed as described by Vasquez et al. (50). Briefly, total RNA was subjected to poly(A) enrichment using a Dynabeads mRNA purification kit (Ambion) followed by an incubation with RNA fragmentation reagent (Ambion). A 15% polyacrylamide gel purification was performed for the size selection (26 to 34 nt) of footprint RNA and fragmented mRNA in comparison with two synthetic RNA markers (IDT [Integrated DNA Technology]).

The mRNA and ribosome footprint sequencing libraries were generated following the protocol of Ingolia et al. (53), except the rRNA depletion steps (steps 47 to 54), which were discarded, and the last amplification step (step 55) was performed using the 2× Kapa HiFi Hot Start Mix (Kapa Biosystems). Libraries were then sequenced using an Illumina NextSeq 500 system.

### Preprocessing and mapping of reads.

For the RiboTaper pipeline (54), cutadapt (95) and Bowtie (96) were used to clip adapters and filter rRNA reads, respectively. Thereafter, the STAR aligner (97) was used to map remaining reads to the genome and the resulting alignment files were sorted and indexed using SAMtools (98). The create_metaplots.bash script from the RiboTaper pipeline was used to generate site coverage plots. The RiboTaper script was then started with appropriate read and cutoff parameters. Alignments of RFP reads and RNA-Seq reads were imported and graphically displayed using the Assemble module of the MacVector software suite and imported into the Intaglio vector graphics software for figure assembly.

To correct for variables due to library preparation efficiency, we normalized the number of protected sites (P-sites) and RNA reads per coding sequence (CDS), respectively, using the median number of reads per CDS for each sample. The translation efficiency (TE) was then calculated from those median-normalized read numbers (TE = P-sites/RNA sites).

To determine changes to protein synthesis, RNA abundance, and translation efficiency, we performed pairwise comparison of P-sites/CDS (RFP = ribosome footprints), RNA sites/CDS (RNA), and TE (Table S5).

### Data availability.

All raw sequencing reads were deposited at the NCBI Sequence Read Archive (SRA) under the project no. PRJNA495919.
