# Supplementary figures and images for: Ribosome Profiling Reveals HSP90 Inhibitor Effects on Stage-Specific Protein Synthesis in Leishmania donovani
Source: mSystems. 2018 Nov 20;3(6):e00214-18. doi: 10.1128/mSystems.00214-18 (PMC6247020; doi:10.1128/mSystems.00214-18)

# IC<sub>80</sub> of Radicicol

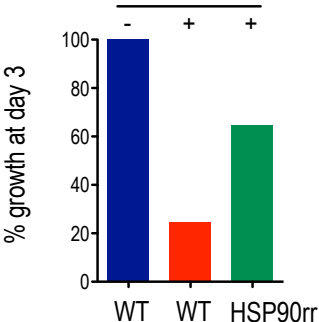

Supplement: FIG S1 [file sys006182293sf1.pdf]

Absorbance at 254 nm

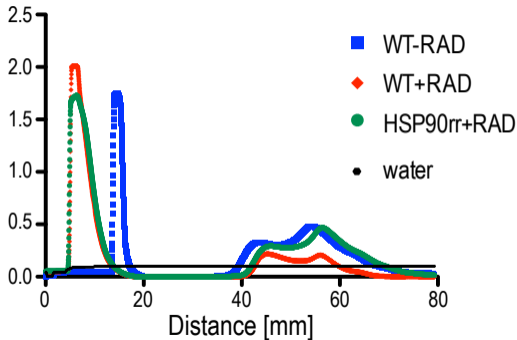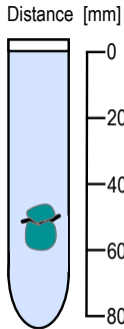

Supplement: FIG S2 [file sys006182293sf2.pdf]
